# Consequences of Cold-Ischemia Time on Primary Nonfunction and Patient and Graft Survival in Liver Transplantation: A Meta-Analysis

**DOI:** 10.1371/journal.pone.0002468

**Published:** 2008-06-25

**Authors:** James E. Stahl, Jennifer E. Kreke, Fawaz Ali Abdul Malek, Andrew J. Schaefer, Joseph Vacanti

**Affiliations:** 1 MGH- Institute for Technology Assessment, Massachusetts General Hospital, Harvard Medical School, Boston, Massachusetts, United States of America; 2 Department of Medicine, Massachusetts General Hospital, Harvard Medical School, Boston, Massachusetts, United States of America; 3 Department of Radiology, Massachusetts General Hospital, Harvard Medical School, Boston, Massachusetts, United States of America; 4 Department of Industrial Engineering, University of Pittsburgh, Pittsburgh, Pennsylvania, United States of America; 5 University of Pittsburgh School of Medicine, Pittsburgh, Pennsylvania, United States of America; 6 Industrial and Management Systems Engineering Department, University of Kuwait, Kuwait City, Kuwait; 7 Department of Pediatric Surgery, Massachusetts General Hospital, Harvard Medical School, Boston, Massachusetts, United States of America; German Cochrane Center, Germany

## Abstract

**Introduction:**

The ability to preserve organs prior to transplant is essential to the organ allocation process.

**Objective:**

The purpose of this study is to describe the functional relationship between cold-ischemia time (CIT) and primary nonfunction (PNF), patient and graft survival in liver transplant.

**Methods:**

To identify relevant articles Medline, EMBASE and the Cochrane database, including the non-English literature identified in these databases, was searched from 1966 to April 2008. Two independent reviewers screened and extracted the data. CIT was analyzed both as a continuous variable and stratified by clinically relevant intervals. Nondichotomous variables were weighted by sample size. Percent variables were weighted by the inverse of the binomial variance.

**Results:**

Twenty-six studies met criteria. Functionally, PNF% = −6.678281+0.9134701*CIT Mean+0.1250879*(CIT Mean−9.89535)
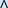
2−0.0067663*(CIT Mean−9.89535)
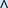
3, r2 = .625, , p<.0001. Mean patient survival: 93 % (1 month), 88 % (3 months), 83 % (6 months) and 83 % (12 months). Mean graft survival: 85.9 % (1 month), 80.5 % (3 months), 78.1 % (6 months) and 76.8 % (12 months). Maximum patient and graft survival occurred with CITs between 7.5–12.5 hrs at each survival interval. PNF was also significantly correlated with ICU time, % first time grafts and % immunologic mismatches.

**Conclusion:**

The results of this work imply that CIT may be the most important pre-transplant information needed in the decision to accept an organ.

## Introduction

Procuring and transplanting organs are the two main functions of the liver allocation system. Preserving livers until they can be transplanted is an essential intermediate step. The U.S. Organ Procurement and Transplantation Network and the Scientific Registry of Transplant Recipients, has recently has begun to publish the effect of cold-ischemia time (the time from clamping of the donor aorta until the anastomosis of the organ to the recipients vascular system or the organs disposal) on clinical outcomes- patient and graft survival [1]. Many other factors also contribute to the success or failure of an organ to function [2], [3], [4], [5], [6], [7], [8], [9], [10], [11], [12] once transplanted and on patient and graft survival. However, cold-ischemia time also determines in part how far we can transport organs [13]. This in turn influences the size of the pool of organs available to patients [14]. The larger the pool the more chances for an optimal donor/recipient match. Therefore, understanding the effectiveness of organ preservation technology is important in understanding how best to allocate organs.

The primary purpose of this project is to help quantify and describe the functional relationship between cold-ischemia time and primary nonfunction (death or retransplant due to liver failure within 14 days of initial transplant unrelated to acute rejection) and patient and graft survival. In addition, we also examined the effect of other factors hypothesized to influence initial graft function and patient and graft survival. This information will help understanding the functional limitations of the liver allocation system and how best to optimize its performance.

## Methods

### Literature search

**Figure 1:**
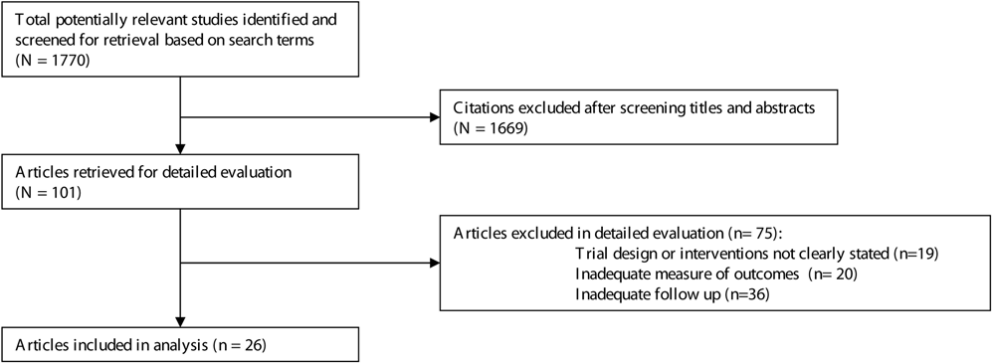
Article selection flowchart.

We performed a search of MEDLINE, EMBASE and the Cochrane database, including the non-English literature identified in these databases, to identify all potentially relevant articles that were published between 1966 and January 2008 that reported the duration of preservation of cadaveric livers and their function after transplant. The search was further augmented by scanning the references of the identified articles and reviews (JES). See Figure 1.

### Assessment of validity

Two separate investigators (JK, FA) independently evaluated studies for inclusion. The observed agreement between the investigators for the assessment for inclusion was calculated using the kappa statistic. After the initial article selection, the article dataset was reviewed and updated to capture any articles published between the final consensus review and the final data analysis (JES). Articles were potentially acceptable if they were randomized-controlled trials, case-control trials with appropriate matching or prospective observational studies. Trials were scored using a quality rating system adapted from Chalmers [15], [16], Cochrane Collaboration [17] and others.

Our scoring system attempted to remove studies with significant selection bias, performance bias, attrition bias or detection bias. Our scoring system included the following questions, 1) Was the trial design clearly stated?, 2) Selection bias questions: Was the Patient selection process clearly stated?, If the trial was an RCT, were patient randomly allocated to the therapeutic intervention? Were patients and clinicians blinded to the intervention? If the trial was not an RCT, were confounders controlled for? If the trial design was case control were matching procedures clearly described and implemented? Were patient recruitment procedures clearly described? Were the intervention and control groups selected for similarly? 3) Performance bias questions: Was the intervention clearly described? , Was intervention clearly measured? 4) Attrition bias questions: Were patients followed up?, Were they followed up for 2 or more explicitly defined intervals? If patients were lost/ dropped out other than due to death were they accounted for? Were all outcome measures captured at the declared follow up intervals? 5) Detection bias questions: Were the outcome measures clearly described? Was measurement of the outcome measures blinded? 6) Were appropriate statistical methods used?, were p values clearly states, was life table analysis provided, etc. 7) Was the presentation of data adequate, for example, in the article were endpoints clearly defined i.e., PNF, graft survival, patient survival, duration of follow up, retransplantation rate, etc? Were survival curves provided or were sufficient data to construct survival curves provided, were donor and recipient variables clearly defined and presented [15], [16].

These questions were placed on a 3 point scale: Unclear/inadequate (0), adequate (1), good (2). Articles were considered for inclusion if their summary score exceeded 30. The kappa statistic for the independent reviewers was .93.

### Study Characteristics

The primary independent variable of interest was the cold ischemia time of the procured cadaveric liver. Studies were stratified by duration of preservation (hours), the preservation method used (Euro-Collins solution, University of Wisconsin solution, Celsior solution, etc.), and the primary outcome of initial graft function (primary nonfunction) and patient and graft survival. Primary nonfunction is defined broadly as death or retransplant due to liver failure within 14 days of initial transplant unrelated to acute immunologically-mediated rejection [18], 19, [20].

### Data extraction

Two independent reviewers extracted data from the selected articles, reconciling any differences by consensus or when in doubt referring it for arbitration by a third reviewer (JES). The mean CIT for each preservation solution subgroup in each of the studies was extracted or calculated from the data provided. CIT was defined as cold perfusion until the time of circulatory reperfusion or disposal.

Other variables hypothesized as having a relationship to initial graft function and patient and graft survival were also extracted. For donors these included: age, gender, race, HLA-matching, biopsy histology, warm ischemia time, organ procurement time, Monoethylglycinexylide (MEGX, a metabolic function test) test results, ICU days, and bilirubin, creatinine, AST and ALT (liver transaminases) levels at the time of procurement. For recipients these included: age, gender, race, weight, pre-transplantation waiting time, previous abdominal surgery, ABO blood group or match/no match with donor, pre-transplant Karnofsky score, infection status, nutritional status, etiology of end-stage liver disease, UNOS and Child-Pugh score and number of previous donor/recipient mismatches. We also extracted peri-operative variables such as the use of a reduced or “split” liver, surgery duration, blood loss, bile flow in the first three days, peak post transplant bilirubin, prothrombin time, ammonia, creatinine, AST and ALT (liver transaminases) levels. Finally, we also extracted the re-transplantation rate within 3 months. Data was tested for heterogeneity using Chi2, and graphically using normal-quantile plot as a test of the normality of the distributions and funnel plots of the variable vs. year of study.

### Data synthesis and analysis

To evaluate the effect of CIT on immediate post-transplant function and patient and graft survival, we first determined the mean duration of CIT for each preservation solution group in the study. We then determined the rate of primary nonfunction (PNF) for each of these groups to estimate the effect of preservation duration on the immediate post-transplant success. We also determined the 1 month, 3 month, 6 month, 1 year, 2 year, 5 year and 10 year patient and graft survival for these groups. For each time point a weighted mean estimate of survival was generated.

Because we hypothesized that the rate of PNF would vary continuously with the duration of CIT [1], [13], we could not expect the sample to be homogenous within any particular subset. We therefore analyzed the mean CIT data both as a continuous function, as well as, stratified by ordinal time intervals hypothesized to be clinically relevant.

Each study reported an intervention and control group. Typically two preservation solutions were compared. Each article reported one or more subgroups with differing mean CITs. We assumed that the recipients of a transplant were essentially the same in with regard to clinical urgency across preservation solutions within articles. In addition, after preliminary inspection of the data it became apparent that very few studies meeting inclusion criteria had subgroups with preservation fluids other than University of Wisconsin (UW) (80%), Euro-Collins (EC) (14.5%) or Celsior (5.5%) as their primary preservation solution, typically a test and control solution. In addition, groups using Celsior or Euro-Collins solutions tended to have shorter CITs. The potential difference in efficacy between solutions independent of CIT was controlled for via regression analysis much in the same way one would control for gender when predicting the relationship between age and weight. To test for any consequences of mixing different designs we conducted further subgroup and sensitivity analyses based on study design.

These study subgroups were further stratified into intervals of CIT. The 5 subgroups based on mean CIT were: <5 hrs, 5–7.5 hrs, 7.5–10 hrs, 10–12.5 hrs, and >12.5 hrs. These intervals are narrower than the 5 hours increments reported in the OPTN/SRTR Annual report [1]. The time intervals chosen were a compromise between inter-quintile ranges of the reported mean CITs and clinical relevance. The reported data beyond 12.5 hours the data becomes progressively more sparse, variable and difficult to provide meaningful analysis on. For each CIT interval, we tested whether or not the effect (PNF, patient and graft survival) was homogenous within the interval using fixed and random effects models [21]. Chi-square tests were used to test the inter-study variability. A p-value less than .1 was considered significant. However, because we hypothesized that PNF and survival would vary with mean CIT, we also analyzed the data using mean CIT as a continuous function. In addition, where possible we stratified donor and recipient variables to test for any significant differences in the subgroups.

The outcomes, PNF and patient and graft survival were estimated as a weighted percents. All three were treated as having binomial distributions since the outcomes were binomial, e.g., survive/ no survive at any given point in time. Therefore, the effect of a given study at a given point in time, e.g., 3 months, was p (percent). Its weight was the inverse of its variance (1/npq) where n is the number in the subgroup and q is 1- p. These outcomes were also at the post transplant intervals of 1 month, 3 months, 6 months and 1 year.

Each subgroup contributed distinct data on its mean CIT, survival and other parameters of interest, which was weighted as described above. The functional relationships between CIT and initial organ function and patient and graft survival were explored through regression analysis of the subgroup data. Summary point estimates of, for example, mean length of stay, were derived by pooling the weighted parameter estimates for each subgroup. Other factors hypothesized as influencing these relationships, for example, age, were tested for significance using standard least square models on the subgroup data.

### Role of the funding source

Funding for this study came from the AHRQ, the NSF, and salary support for graduate students at University of Pittsburgh. Neither the Massachusetts General Hospital nor the University of Pittsburgh had any role in either the design, conduct or reporting of the study. None of the authors had any conflicts of interests in this study.

## Results

### Trial characteristics

There were 26 studies included in the final analysis: 4 randomized controlled trials, 6 prospective cohort studies and 16 case-control studies. The earliest was published in 1989. Table 1 shows the characteristics of the studies included in the meta-analysis. The quality of all included trials quality was either adequate [1], [3], [5], [6], [19], [22], [23], [24], [25], [26], [27], [28], [29], [30], [31], [32], [33], [34], [35], [36], [37], [38], [39] or good [40], [41], [42]. The studies included (Table 2) patients of which 64.3% of the donors and 54.1% of recipients were male. The mean age for donors was 29.9 years (s.d. = 1.3) and mean age of the recipients was 40.6 years (s.d. = 8.6). The mean study subgroups sample size was 99 (s.d. = 116) and median patient follow up was 12 months (range 1–72 months) median graft follow up was 12 months (range 1–60 months). No study reported the full set of all donor variable and recipient variables identified as being of possible importance. Table 3 shows the variables collected where more than one study reported results. Not all studies provided parameter variance estimates. Therefore, nondichotomous variables were weighted by sample size. Outcome variables reported as percents were weighted by the inverse of the binomial variance, i.e., 1/npq.

**Table 1:** Study design and preservation subgroups.

	Study design			
	RCT [40], [41], [42], [57]	Prospective observational cohort [1], [5], [6], [25], [28], [38]	case control [3], [5], [19], [22], [23], [24], [29], [31], [32], [33], [34], [35], [36], [37], [39], [58]	Total
Studies	4	6	16	26
	Preservation subgroup samples			
UW	4	7	33	44
EC	0	0	8	8
Cel	3	1	0	4
				56

**Table 2 pone-0002468-t002:** Study size and demographic characteristics.

	Min	Med	Max
Study size (n)	30	173	710
Subgroup size (n)	17	52	710
Donor Age (years)	19.6	29.5	54.9
Donor Gender M:F	1	1.8	2.8
Recipient Age (years)	23.5	43	54
Recipient Gender M:F	0.7	1.2	2
UNOS 1 (%)	6.8	28	36.6

**Table 3:** Weighted mean values for donor and recipient variables.

Variable	Weighted Mean	S.d.	References
**Donor variables**			
Mean Warm Ischemia time (hours)	1.14	0.04	[24], [25], [32], [41], [42], [58]
Mean Length of stay (days)	3.2	0.17	[3], [22], [29], [35], [59]
Mean ICU stay (days)	3.36	0.07	[19], [41], [42], [57]
Race (Caucasian) %	98	1.5	[3], [6], [33]
Race (Non Caucasian) %	2	0.7	[3], [6], [33]
**Recipient lab variables during index admission**			
Percent ABO Donor /recipient mismatch	2.1	0.8	[29], [37]
Peak serum Bilirubin	81.5	12.1	[22], [23], [24], [29], [32], [33], [35], [38], [39], [57], [58]
Mean AST (index admission)	456.6	84.4	[22], [24], [39]
Peak AST (index admission)	1349	89.8	[23], [24], [28], [29], [32], [35], [36], [37], [38], [39], [40], [57], [58], [59]
Peak PT (index admission)	37.7	2.5	[22], [24], [35], [57], [58]
Peak ALT (index admission)	979.4	51.1	[22], [23], [24], [32], [33], [35], [36], [37], [38], [39], [40], [57], [59]
**Recipient health status**			
UNOS status 1 (%)	25.7	3.1	[5], [25], [38], [42]
UNOS status 2 (%)	43.6	12.3	[5], [25], [32], [38], [42], [57]
UNOS status 3 (%)	21.4	11.4	[1], [5], [25], [32], [38], [42], [57]
UNOS status 4 (%)	26.6	1.4	[29], [32], [38]
UNOS status 1 or 2 (%)	45.9	4.5	[1], [5], [6], [25], [32], [38], [42], [57], [58]
UNOS status 3 or 4 (%)	50.3	3.7	[5], [6], [25], [29], [32], [37], [38], [42], [57], [58]
**Recipient ESLD Diagnosis at Transplant**			
Fulminant liver failure	10	2.5	[5], [6], [25], [26], [33], [34], [35], [37], [41], [59]
HCVorHBV	17.3	7.7	[6], [24], [25], [33], [34], [37], [41], [59]
Alcohol related ESLD	10.3	2.2	[6], [25], [26], [33], [34], [41], [59],
Primary biliary cirrhosis	23.3	9.2	[5], [24], [26], [33], [34], [35], [37], [59]
Primary sclerosing cholangitis	5.8	2.5	[33], [34], [35], [37], [59]
AutoImmune-related ELSD	2.9	1.2	[25], [26], [33], [34]
Metobolic-related ESLD	7.2	1.6	[5], [6]
Cryptogenic ESLD	12.6	3.6	[26], [33], [34], [37], [59]
Neoplastic-related ESLD	5.7	5.3	[6], [22], [26], [33], [34], [35], [37]
Other ESLD	7.2	3.4	[5], [6], [22], [25], [26], [33], [34], [35], [41], [59]
**Recipient hospital variables**			
Mean ICU LOS (days)	8.4	0.2	[26], [57], [58]
Mean Hospital LOS (days)	34.3	1.3	[25], [26], [34], [35], [58]
Re-transplant within 30 days of index admission	6.3	2.6	[1], [2], [5], [6], [19], [22], [23], [24], [25], [26], [28], [29], [32], [33], [34], [35], [36], [37], [38], [39], [40], [41], [42], [58] [59]

Using chi statistic to test for heterogeneity of PNF within the CIT intervals, we find there is low heterogeneity for intervals under 7.5 hours and increasing heterogeneity and fewer studies as we move to the tail of the PNF vs CIT distribution: For the CIT interval 0–2.5 hrs the p value for Chi2 was .95 and I2 was 24.9, for the CIT interval 2.5–5 hrs the p value for Chi2 was .95 and I2 was 36.6, for the CIT interval 5–7.5 hrs the p value for Chi2 was .95 and I2 was 36.6, for the CIT interval 7.5–10 hrs the p value for Chi2 was .94 and I2 was 81, for the CIT interval 10 > hrs the p value for Chi2 was .94 and I2 was 95.6.

There were no significant difference across the predetermined CIT intervals with regard to donor gender (p = .9), warm ischemia time (time from last artery clamped to immersion in preservation solution) (p = .47), MEGX score (p = .9 ), recipient age (p = .66), recipient gender (p = .68), or recipient AST peak within index admission (p = .54), length of stay (p = .18) in the hospital or ICU (p = .15). In addition, there were no significant differences across these intervals with regard to UNOS score. However if you grouped by UNOS 1 and 2 there was a significant trend (p = .01, r2 = .9 ) toward increase urgency the higher the CIT. IN addition there was significant trend for increasing recipient bilirubin peak (p = .06, highest for CIT between 5–10 hrs) and recipient PT peak (p = .01, 2 = .7) the longer CIT. However, as predicted (the relationships between CIT and PNF and patient and graft survival were hypothesized as being continuous), PNF and patient and graft survival varied systematically with CIT. There was significant difference across these intervals with regard to PNF and patient and graft survival (see figure 2–6).

**Figure 2:**
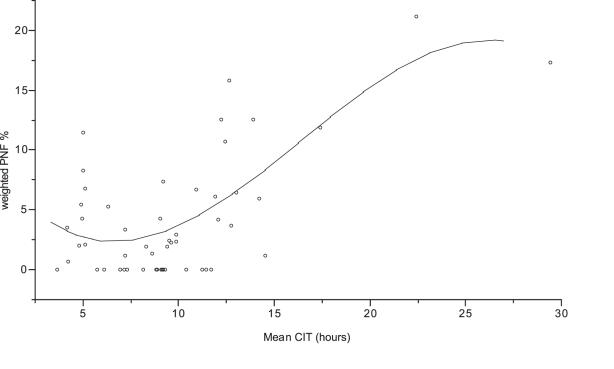
Weighted PNF (%) vs. mean CIT for each study subgroup. PNF% = −6.678281+0.9134701*CIT Mean+0.1250879*(CIT Mean−9.89535)
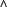
2−0.0067663*(CIT Mean−9.89535)
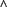
3. r2 = .625. p = <.0001.

**Figure 3 pone-0002468-g003:**
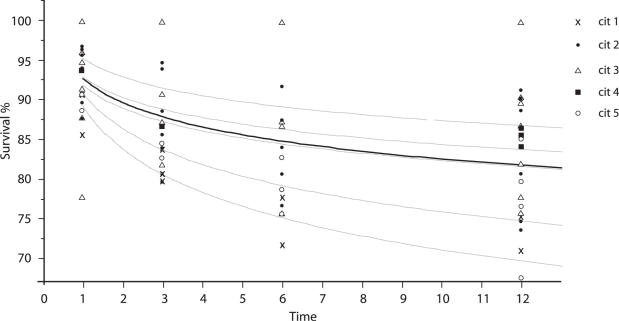
Patient survival stratified by CIT interval. Cold-ischemia time (CIT) intervals: cit 1 (<5 hrs), cit 2 (5–7.5 hrs), cit 3 (7.5–10 hrs), cit 4 (10–12.5 hrs), and cit 5 (>12.5 hrs.). Time units = months.

**Table:** 

Total fit	survival = 92.378882−3.9346785*Log(time)	r2 = .27	p<.0001
Cit1	Patient Survival (%) = 89.02784−6.7750233*Log(time)	r2 = .86	p = .0001
Cit2	Patient Survival (%) = 94.782189−4.3027938*Log(time)	r2 = .30	p = .0003
Cit3	Patient Survival (%) = 92.007176−2.3023009*Log(time)	r2 = .1	p = .145
Cit4	Patient Survival (%) = 92.650084−2.9404238*Log(time)	r2 = .8	p = .04
Cit5	Patient Survival (%) = 91.687229−5.2662024*Log(time)	r2 = .52	p = .002

**Figure 4 pone-0002468-g004:**
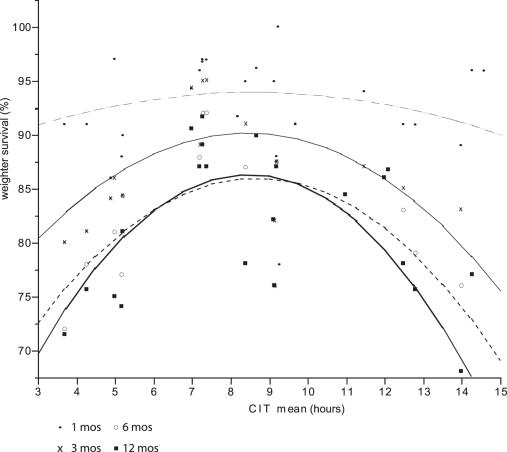
Patient Survival at 1, 3, 6 and 12 months versus mean CIT.

**Table:** 

Total	Patient Survival (%) = 85.312258+0.5717633*cit mean−0.3088593*(cit mean−8.47705) 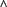 2	r2 = .17	p = .0006
At 1 month	Patient Survival (%) = 91.489057+0.1840028*cit mean−0.0454594*(cit mean−8.47705) 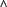 2	r2 = .02	p = .83
At 3 month	Patient Survival (%) = 89.178376+0.3534126*cit mean−0.4544172*(cit mean−8.47705) 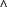 2	r2 = .51	p = .006
At 6 month	Patient Survival (%) = 84.856021+0.5231569*cit mean−0.5752957*(cit mean−8.47705) 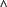 2	r2 = .54	p = .0095
At 12 month	Patient Survival (%) = 78.211314+1.0064161*cit mean−0.3878469*(cit mean−8.47705) 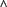 2	r2 = .29	p = .024

**Figure 5 pone-0002468-g005:**
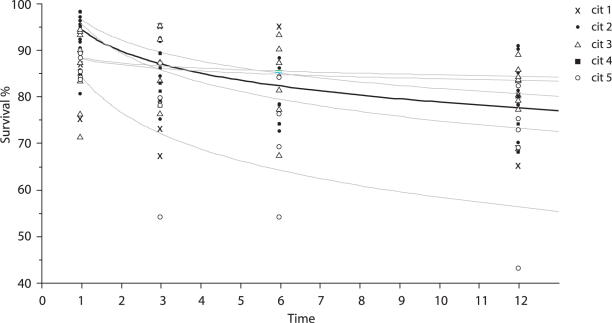
Graft survival stratified by CIT interval. Cold-ischemia time (CIT) intervals: cit 1 (<5 hrs), cit 2 (5–7.5 hrs), cit 3 (7.5–10 hrs), cit 4 (10–12.5 hrs), and cit 5 (>12.5 hrs.).

**Table:** 

Total	Graft Survival (%) = 89.026051−4.9055276*Log(Time)	r2 = .24	p = <.0001
Cit1	Graft Survival (%) = 88.704349−6.1987377*Log(Time)	r2 = .25	p = .079
Cit2	Graft Survival (%) = 91.657328−4.8387205*Log(Time)	r2 = .38	p = <.0001
Cit3	Graft Survival (%) = 87.625028−2.1579358*Log(Time)	r2 = 09	p = .11
Cit4	Graft Survival (%) = 92.182039−7.0580881*Log(Time)	r2 = .72	p = .0001
Cit5	Graft Survival (%) = 84.720783−6.9963426*Log(Time)	r2 = .3	p = .0096

**Figure 6:**
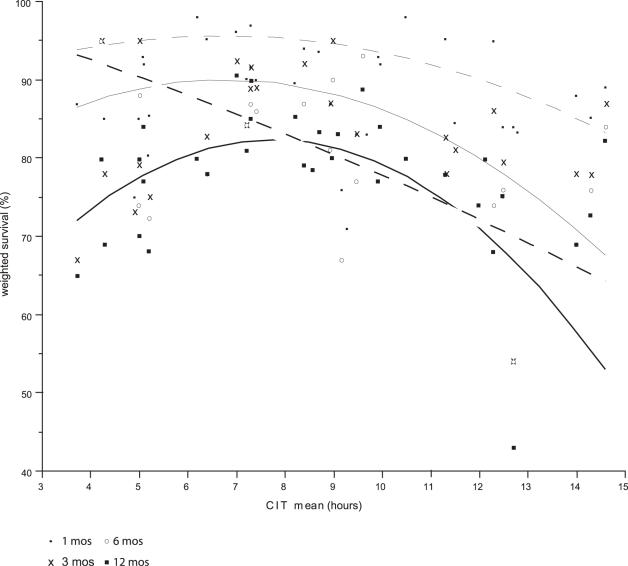
Graft Survival at 1, 3, 6 and 12 months versus mean CIT.

**Table:** 

Total	Graft Survival (%) = 87.665963−0.2954854*CIT Mean−0.2668728*(CIT_Mean−8.78025) 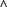 2	r2 = .1	p = .003
At 1 month	Graft Survival (%) = 89.256426+0.0114638*CIT Mean−0.1162638*(CIT_Mean−8.78025) 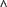 2	r2 = .03	p = .64
At 3 month	Graft Survival (%) = 87.88981−0.2009686*CIT Mean−0.3122215*(CIT_Mean−8.78025) 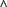 2	r2 = .13	p = .19
At 6 month	Graft Survival (%) = 90.874959−1.1789163*CIT Mean−0.0410205*(CIT_Mean−8.78025) 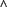 2	r2 = .17	p = .18
At 12 month	Graft Survival (%) = 83.977982−0.2318567*CIT Mean−0.4397392*(CIT_Mean−8.78025) 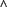 2	r2 = .24	p = .019

As mention above, the subgroups using EC or Celsior solutions did have significantly shorter mean CIT (6 hrs) than the UW subgroups (10 hrs). However, the adjusted PNF for the EC or Celsior groups were not significantly different from the UW group nor were they significantly different on any of the contextual variables listed in Tables 1–3 when controlled for by CIT. The one exception was for those groups with a CIT>12.5 hrs. For this group the rate of blood type mismatch (p = .05) and recipient mean AST during index admission (p = .03) were significantly worse than for other CIT intervals. There were insufficient studies to demonstrate whether or not the PNF rate for those using UW (3%) with a CIT shorter than 6 hours was not significantly different than the EC and Celsior (4%) groups (p = .37).

Mean CIT did not significantly vary by year of publication. However, both average donor age (1.67 yrs per year, p<.0001) and recipient age did increase significantly (.0 years per year, p = .004) over the period of time (1989–2007) covered by the studies. There was a trend towards reduced PNF over this period but this did not reach the level of significance (0 = .46) Subgroup analysis and sensitivity analyses did not reveal any significant difference in the results of the analyses across study designs (p = .17), however, the trend appeared to be for RCTs to have lower PNFs.

Across all groups, PNF was not significantly correlated with recipient ICU time (p = .8), inversely related with the percentage of first time grafts transplanted (p = .3) and positively correlated related with the percent of ABO mismatches (p = .56).

### Regression analysis and Quantitative data synthesis

#### PNF

For the whole group the mean PNF was 7.8% (s.d. = 9.3%) and the median PNF was 2% (range 0–21.2 %). PNF varied systematically with CIT. A third degree function relating mean CIT with weighted PNF had an r2 = .55, p = <.0001, which is significantly better than linear, second degree or natural log forms. This function was:

Weighted PNF% = −0.045838+0.0070228 mean CIT+0.0015596 (mean CIT−9.63524)
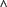
2−0.0000771 (mean CIT−9.63524)
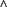
3, r2 = .625, p<.0001. See Figure 2. Univariate analysis indicated that other than mean CIT, PNF was directly proportional to recipient LOS (p = .13).

### Patient Survival

Mean patient survival for the whole group was 93 % at 1 month, 88 % at 3 months, 83 % at 6 months and 83 % at 12 months. An exponential patient survival function was assumed. This was % Survival = 93.18−4.4 Log(time) for the whole group. See Figure 3. If one regressed patient survival against CIT at 1 month, 3 months, 6 months, and 12 months post-transplant, a quadratic relationship with CIT is revealed, with survival reaching a maximum with CITs between 7.5 and 10 hours. Patient survival worsens above and below this interval. See Figure 4.

### Graft Survival

Mean graft survival for the whole group was 85.9 % at 1 month, 80.5 % at 3 months, 78.1 % at 6 months and 76.8 % at 12 months. An exponential survival function was assumed. This was % Survival = 85.29−3.59 Log(time) for the whole group. See Figure 5. If one regressed graft survival against CIT at 1 month, 3 months, 6 months, and 12 months post-transplant, a quadratic relationship with CIT is also revealed. As with patient survival, maximum survival at each of these post transplant time points reached a maximum when the graft's CIT was between 7.5 and 12.5 hours. Graft survival worsens above and below this interval. See Figure 6.

## Discussion

Cold-ischemia time appears to be a good predictor of not only PNF but also of patient and graft survival [1], [13]. The damage to livers incurred during prolonged CIT has been hypothesized as being the cause of PNF. This in turn has been hypothesized to result from injury to the hepatic sinusoidal epithelial cells, which in turn results in a cascade of injuries involving the microcirculation and the release of various cytotoxic products [43], [44], [45]. The resulting damage to the hepatocytes presumably results in varying levels of organ dysfunction up to and including primary hepatic nonfunction at transplant. This may be exacerbated by other factors such as warm-ischemia time [13].

Cold-ischemia time along with the supply of organs, patient demand and the matching regime appear to be the key parameters to the liver allocation system. Therefore, reducing CIT through more efficient organ allocation [13], [14] or improving the preservation method should improve outcomes and the effective use of a limited healthcare resource. Understanding the relationship between CIT, PNF and patient and graft survival can help better define the limits and needs of our allocation system and better match more organs to patients.

A key factor determining the consequences of CIT is the preservation method. Preservation methods have evolved and improved considerably over time and may be expected to continue to do so. The better preservation becomes the closer the system resembles banking systems like the one used for blood products. Such a system would allow better buffering of imbalances between supply and demand. Historically, the first preservation solutions were isotonic electrolytic solutions for skin grafts such as Ringer's solutions in the 1890s [46]. Refrigeration came into use in the 1940–50s to preserve skin used as temporary dressing for burns (www.transweb.org/reference/timeline). Since the first successful solid organ transplants (Kidney 1953 Boston, JE Murray, Brigham and Women's Hospital, Boston, MA) and the first successful liver transplant (Liver 1963, Denver, T Starzl, Univ. Col.) [21], there has been a growing need to be able to preserve organs for longer periods of time. The most commonly used preservation media have been Euro Collins [47], University of Wisconsin Solution [48] and Celsior solution [49]. At first cut, in our study groups using Celsior solution appeared to perform better with regard to PNF than the other groups. However, the Celsior patients also had shorter CITs than either the UW or EC groups. Although there appeared to be the beginnings of a trend indicating improved outcomes with Celsior, we had insufficient studies using Celsior solution, due to the strict constraints of our inclusion and exclusion criteria, to determine if this solution performed better when controlling for CIT. The results presented in this study represent an aggregation of the performance of preservation solutions to date. This study cannot capture the behavior preservation technologies currently in trial.

In addition to CIT and preservation solution, many other donor and recipient factors have been hypothesized as contributing to poor outcomes [4], [5], [8], [9], [38], [50], [51], [52], [53], [54], [55]. Being able to use a simple blood test or patient historical factor to predict outcome prior to transplant has obvious advantages for making organ allocation decisions. The only factors, other than CIT, we found to consistently and significantly associated with poor outcome were: the recipient ICU length of stay after transplant, blood type incompatibility between graft and host, and the percentage of first time transplant recipients in the group. We might hypothesize that these factors are more strongly associated with sicker (e.g., longer ICU stays) and/or more desperate patients (e.g., willing to accept a blood type mismatch). While no individual lab test rose to the level of significance in predicting PNF, it remains possible that combinations of these lab tests might be predictive where individually they are not. With regard to the final item, we might hypothesize that first time transplant recipients may be more clinically urgent or more likely to have an undiagnosed immuno-incompatability than repeat transplant patients.

The relationship between CIT and PNF also appears not to be strictly linear. PNF and organ and patient survival were worse for both high and low CITs. Similar findings are reported in the OPTN annual report [1]. One hypothesis is that this may be a result of disadvantageous combinations of patients and organs. It may be that patients who receive organs less than 5 hours from harvest and those that receive them after 12.5 hours from harvest are more likely to be sicker than average, more clinically urgent or more difficult to find an adequate match. It may be that the freshest organs are rushed to the very sickest patients or that the most desperate patients or the most difficult to match patients with limited options were offered older organs as being the only ones available. It seems that there is a nonlinear interaction between patient and organ offer that determines survival and that this effect is worse when both patient and organ are near the end of their lives. A patient in poor health with a fresh organ results in a slightly worse result than a good organ in a stable patient. Older organs and patients in poor health result in the worst outcomes. Though the data did not reach significance, it is a partial validation of this hypothesis that the greatest percentage of UNOS 1 patients were found in groups with a mean CIT less than 5 hours and >12.5 hours. This may be why those on the low end of CIT seemed to experience only slightly increased PNF and those on the high end experience very significant PNF. Another hypothesis is that this behavior at the extremes of CIT may be related to the characteristics of the regions in which the organs were procured and transplanted. For example, it may be that the quality of the procured organ and the clinical urgency of the persons receiving the transplant may be related to the size of the region in which the transplant took place [56]. Unfortunately, the available data did not provide sufficient information on the site of transplant to reliably test this hypothesis.

Whereas it would be ideal to run a randomized clinical trial to evaluate the question of the effect of cold ischemia time on clinical outcome, this would be impossible because of concerns of ethics and logistic feasibility. It is also very difficult to conduct randomized controlled trial in a surgical setting do to difficulty in blinding participants, hence the need for a meta-analysis to examine this question. However, it should be noted that any meta-analytic exercise runs the risk of publication bias. We checked for this by testing whether or not the extracted data was skewed in one direction or another within the analyzed time strata. Fortunately, no obvious bias was revealed. However, this does not necessarily preclude missing data rather just that the data we do have is self-consistent. We tried to carefully control the quality of the studies we included by setting stringent criteria to exclude problems with selection bias, performance bias, attrition bias, and detection bias as per the consort criteria.

Our analysis seems to indicate that investing in more research into preservation methods would have a clear benefit on organ supply and survival. It may be also possible to create relatively simple decision support tools, based primarily on the CIT of a procured organ, for deciding whether or not to accept that organ for transplant. Thus the results of this research might help improve decision making in the organ allocation system [14].
